# Lung squamous cell carcinoma with hemoptysis after vaccination with tozinameran (BNT162b2, Pfizer‐BioNTech)

**DOI:** 10.1111/1759-7714.14179

**Published:** 2021-10-06

**Authors:** Toshiyuki Sumi, Yuta Nagahisa, Keigo Matsuura, Motoki Sekikawa, Yuichi Yamada, Hisashi Nakata, Hirofumi Chiba

**Affiliations:** ^1^ Department of Pulmonary Medicine Hakodate Goryoukaku Hospital Hokkaido Japan; ^2^ Department of Respiratory Medicine and Allergology Sapporo Medical University School of Medicine Sapporo Japan

**Keywords:** hemoptysis, lung cancer, tozinameran, vaccine

## Abstract

A 66‐year‐old man with squamous cell carcinoma had been receiving chemoradiation therapy after stereotactic radiotherapy for brain metastases. Atezolizumab was initiated as second‐line therapy, after which the patient became progression‐ and recurrence‐free. Four days after his second dose of tozinameran (BNT162b2, Pfizer‐BioNTech), the patient developed persistent hemoptysis. The patient had no thrombocytopenia or coagulation abnormalities. Bronchoscopy revealed active bleeding from the left lingual tracheal branch. The patient was intubated and admitted to the intensive care unit because of increased bleeding. Subsequently, left bronchial artery embolization was performed using a Serescue. Hemostasis was achieved after the procedure, and the patient was discharged 7 days after the onset of hemoptysis. Vaccination against coronavirus disease has been reported to be associated with thrombosis and cerebral hemorrhage, and the hemoptysis in this case was suspected to be induced by vaccination. In summary, the benefits of vaccination exceeded the risks of adverse events in a patient with cancer. However, in conditions such as after chemoradiation, especially in patients with radiation pneumonitis wherein the vasculature is vulnerable, patients should be carefully monitored for hemorrhagic events after vaccination.

## Graphical abstract

Patients in conditions such as after chemoradiation, especially in patients with radiation pneumonitis wherein the vasculature is vulnerable, should be carefully monitored for hemorrhagic events after vaccination.

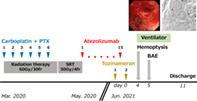

## INTRODUCTION

### Case report

**FIGURE 1:**
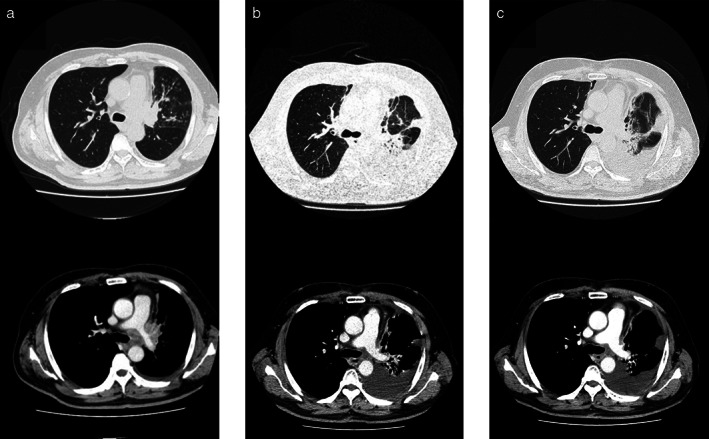
Computed tomography (CT) findings. (a) Before chemoradiation therapy, a mass was detected in the left lung hilum. (b) CT during atezolizumab treatment revealed shrinking of the left hilar mass. (c) CT of hemoptysis with no exacerbation of the left hilar tumor

**FIGURE 2:**
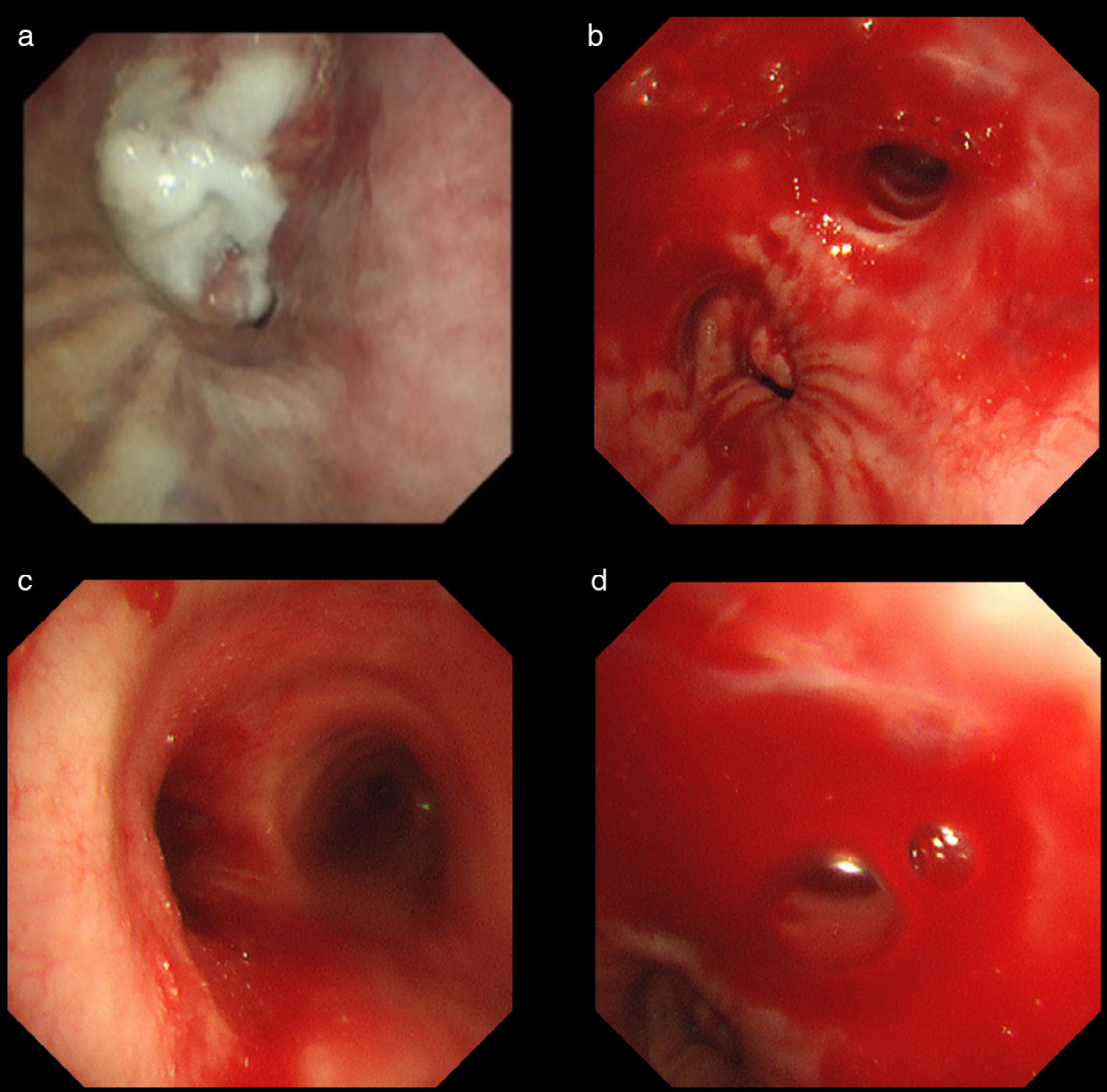
Bronchoscopy findings. (a) Before chemoradiation therapy, the tumor with a necrotic substance on the surface was exposed from the left upper lobe bronchus. (b)–(d) Bronchoscopy for hemoptysis (b) Blood was spilled from the left main bronchus into the tracheal bifurcation. (c),(d) The tumor in the left upper lobe bronchus disappeared. However, active bleeding was observed from the deep bronchus

**FIGURE 3:**
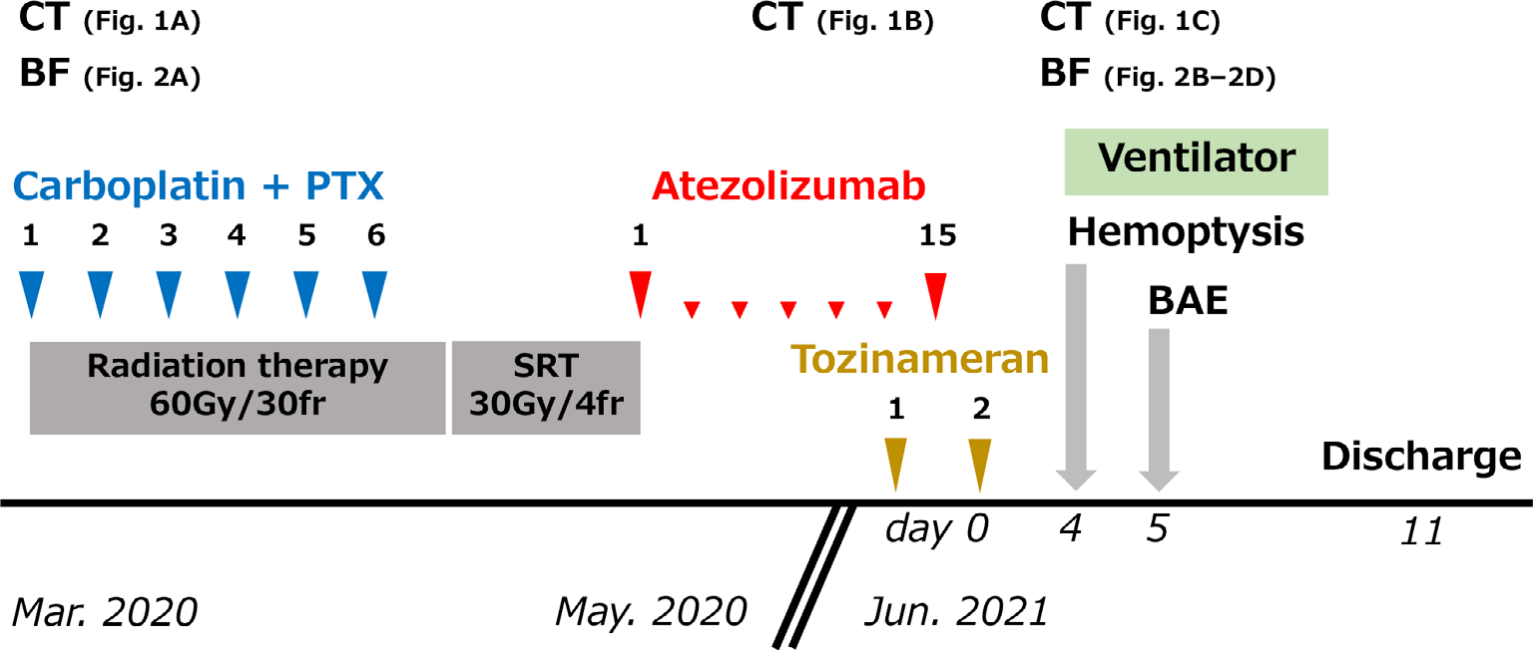
Clinical course. SRT, stereotatic radio therapy; BAE, bronchial artery embolization

A 66‐year‐old man with a squamous cell carcinoma of the hilum of the left lung (cT4N2M0, stage IIIB, programmed death ligand 1 tumor proportion score of 80%) had been receiving chemoradiation therapy since March 2020 (Figure 1(a)). Chemotherapy comprised of carboplatin (area under curve 2) and paclitaxel (40 mg/m^2^) was administered on days 1, 8, and 15 every 3 weeks for 2 cycles. Daily thoracic radiotherapy was administered at a total dose of 60 Gy, 5 days a week in 30 fractions. After chemoradiotherapy, the tumor regressed. However, a solitary 7‐mm‐sized metastatic brain lesion was found in the left frontal lobe, and four fractions of stereotactic radiotherapy (30 Gy) were administered. Atezolizumab (1500 mg/body every 3 weeks) was initiated as second‐line therapy in May 2020. Subsequently, the primary tumor shrank mildly, and no brain metastasis recurrence was observed. The patient became progression‐ and recurrence‐free (Figure 1(b)). Four days after his second dose of tozinameran (BNT162b2, Pfizer‐BioNTech) in June 2021, the patient developed persistent hemoptysis (Figure 1(c)). He had a platelet count of 243 000/mm^3^, prothrombin time and international normalized ratio of 0.96, activated partial thromboplastin clotting time of 26.3 seconds, and D‐dimer levels of 3.0 mcg/mL. Tests for perinuclear anti‐neutrophil cytoplasmic antibodies and perinuclear anti‐neutrophil cytoplasmic antibodies were negative. He had normal levels of platelet‐associated immunoglobulin G and heparin antibodies (Table 1). The patient had been taking aspirin (100 mg/day) for 3 years because of a history of stroke. He discontinued the medication after the hemoptysis onset. In addition, he had been taking amlodipine (5 mg/day) for 10 years for hypertension. Nevertheless, his blood pressure was stable (at ~120/70 mm Hg) and did not increase during hemoptysis. Bronchoscopy revealed active bleeding from the left lingual tracheal branch (Figure 2). The patient was intubated and admitted to the intensive care unit because of increased bleeding, triggered by coughing during bronchoscopy. Subsequently, left bronchial artery embolization was performed using a Cerescue. Hemostasis was achieved after the procedure, and the patient was discharged 7 days after the onset of hemoptysis (Figure 3).

**TABLE 1:** Laboratory data on admission

Hematology	
WBC	6400/μL
Neut	55.7%
Lymph	32.5%
Mono	7.3%
Baso	0.3%
Eosino	4.2%
RBC	434 × 10^4^/μL
Hb	14.4 g/dL
PLT	24.3 × 10^4^/μL
Biochemistry	
TP	7.5 g/dL
Alb	3.5 g/dL
T‐Bil	0.5 mg/dL
AST	35 IU/L
ALT	39 IU/L
ALP	65 IU/L
r‐GTP	83 IU/L
LDH	217 IU/L
CK	79 IU/L
BUN	27.7 mg/dL
CRE	1.33 mg/dL
eGFR	42.7 ml/min/1.73 m^2^
Na	139 mEq/L
K	4.3 mEq/L
Cl	102 mEq/L
CRP	0.15 mg/dL
Coagulation	
PT‐INR	0.96
APTT	26.3 s
D‐dimer	3 μg/mL
Serology	
C‐ANCA	<1.0
MPO‐ANCA	<1.0
PAIgG	36 ng/10^7^cells
PF4	19 ng/mL
SCC antigen	1.2 ng/mL

Abbreviations: C‐ANCA, cytoplasmic antineutrophil cytoplasmic antibody; MPO‐ANCA, myeloperoxidase‐anti‐neutrophil cytoplasmic antibody; PAIgG, platelet‐associated IgG; PF4, platelet factor 4; SCC, squamous cell carcinoma.

## DISCUSSION

The AstraZeneca vaccine (ChAdOx1nCoV‐19) has been reportedly associated with heparin antibody production, thrombosis, and thrombocytopenia.^1^, ^2^ Similar occurrences were observed with the other coronavirus disease (COVID‐19) vaccines. According to the European database, serious adverse events related to thrombocytopenia, bleeding, and thrombosis were observed with the administration of four per one million doses of BNT162b2 and 30 per one million doses of ChA.^3^ Deaths from intracranial hemorrhage (ICH) following Pfizer‐BioNTech vaccination were reported in Japanese women. This indicated that the mortality rate from ICH was disproportionately higher than that reported in the national statistics, suggesting an association between ICH and the vaccine.^4^

In this patient, hemoptysis, and not ICH, occurred after vaccination. Regarding radiotherapy for lung cancer and its effect on blood vessels, radiotherapy for metastatic brain tumors suppresses angiogenesis and normalizes the tumor vascular structure, therefore reducing the risk of bleeding.^5^ Radiation therapy to the lung is associated with marked endothelial cell damage in the pulmonary arteries and a decrease in the vascular bed of the pulmonary arterial system. However, proliferation of the bronchial arteries might occur in patients with radiation pneumonitis.^6^ In this patient, chest irradiation was performed simultaneously with chemotherapy. It is also known that paclitaxel inhibits angiogenesis.^7^ Therefore, the patient was expected to have immature bronchial arteries, and therefore, a high risk for bleeding. Thrombocytopenia was not evident in this patient. However, bleeding possibly occurred from the bronchial artery, which was vulnerable after chemoradiation therapy. It was difficult to perform a biopsy of the bleeding site during bronchoscopy because of the active bleeding. However, during treatment, the tumor did not progress, and hemoptysis did not develop, suggesting that the vaccine was the most likely trigger. The patient's risk factors for severe COVID‐19 included age and medical history of malignancy and chronic obstructive pulmonary disease.^8^, ^9^, ^10^ The American Society of Clinical Oncology and the European Society for Medical Oncology have recommended vaccination of patients with cancer unless there are contraindications such as allergic reactions.^11^, ^12^ In particular, patients receiving immune checkpoint inhibitors should be vaccinated.^12^, ^13^ In summary, the benefits of vaccination exceed the risks of adverse events in patients with cancer. However, in conditions such as after chemoradiation, especially in patients with radiation pneumonitis wherein the vasculature is vulnerable, because there are no useful predictive markers for bleeding events after vaccination, the presence of blood sputum and the respiratory status of the patient should be monitored carefully to detect any event early.

## CONFLICT OF INTEREST

The authors have no conflict of interest.

## PATIENT CONSENT STATEMENT

Consent has been obtained from the patient for reporting this case.
